# Multidecade Mortality and a Homolog of Hepatitis C Virus in Bald Eagles (*Haliaeetus leucocephalus*), the National Bird of the USA

**DOI:** 10.1038/s41598-019-50580-8

**Published:** 2019-10-18

**Authors:** Tony L. Goldberg, Samuel D. Sibley, Marie E. Pinkerton, Christopher D. Dunn, Lindsey J. Long, LeAnn C. White, Sean M. Strom

**Affiliations:** 1 Department of Pathobiological Sciences, University of Wisconsin-Madison, Madison, WI 53706 USA; 2 Global Health Institute, University of Wisconsin-Madison, Madison, WI 53706 USA; 3 Wisconsin Department of Natural Resources, Madison, WI 53707 USA; 4 U.S. Geological Survey National Wildlife Health Center, Madison, WI 53711 USA

**Keywords:** Ecology, Evolution, Genetics, Microbiology, Molecular biology, Zoology, Diseases

## Abstract

The bald eagle (*Haliaeetus leucocephalus*) once experienced near-extinction but has since rebounded. For decades, bald eagles near the Wisconsin River, USA, have experienced a lethal syndrome with characteristic clinical and pathological features but unknown etiology. Here, we describe a novel hepacivirus-like virus (*Flaviviridae*: *Hepacivirus*) identified during an investigation of Wisconsin River eagle syndrome (WRES). Bald eagle hepacivirus (BeHV) belongs to a divergent clade of avian viruses that share features with members of the genera *Hepacivirus* and *Pegivirus*. BeHV infected 31.9% of eagles spanning 4,254 km of the coterminous USA, with negative strand viral RNA demonstrating active replication in liver tissues. Eagles from Wisconsin were approximately 10-fold more likely to be infected than eagles from elsewhere. Eagle mitochondrial DNA sequences were homogeneous and geographically unstructured, likely reflecting a recent population bottleneck, whereas BeHV envelope gene sequences showed strong population genetic substructure and isolation by distance, suggesting localized transmission. Cophylogenetic analyses showed no congruity between eagles and their viruses, supporting horizontal rather than vertical transmission. These results expand our knowledge of the *Flaviviridae*, reveal a striking pattern of decoupled host/virus coevolution on a continental scale, and highlight knowledge gaps about health and conservation in even the most iconic of wildlife species.

## Introduction

For its majestic splendor, the bald eagle (*Haliaeetus leucocephalus*) was selected as the emblem of United States of America by the Continental Congress of 1782^1^. For millennia prior, the bird was recognized as sacred by diverse Native American peoples^2^. Despite its importance to North American histories and cultures, the bald eagle nearly went extinct in the 1960s due to eggshell thinning caused by dichlorodiphenyldichloroethylene (p,p’-DDE), a biodegradation product of dichlorodiphenyltrichloroethane (DDT)^3,4^. The publication of Rachel Carson’s *Silent Spring* in 1962^5^, subsequent banning of agricultural DDT use in the USA and Canada in the early 1970s^6^, and improved protections under the Bald and Golden Eagle Protection Act of 1940 and the Endangered Species Act of 1973^7,8^ helped the species recover from a nadir of 487 breeding pairs in the coterminous United States in 1963 to over 16,000 breeding pairs in 2009^9–11^. The bald eagle was removed from the US federal list of threatened and endangered species in 2007^7^. Currently, major causes of bald eagle mortality, especially for birds living near people, include poisoning, trauma, electrocution, and illegal hunting^12,13^.

Infections of bald eagles have been documented since the early 1900s but (with the possible exception of West Nile virus^14,15^) have generally been considered sporadic and incidental^10,12,16,17^. Among 2,980 bald eagle carcasses submitted to the U.S. Geological Survey National Wildlife Health Center (NWHC) in Madison, Wisconsin, USA, between 1982 and 2013, only 5.2% of deaths were attributed to infectious diseases^12^. Documented infectious diseases of bald eagles include: ectoparasitoses^18,19^, helminthoses^20–24^, aspergillosis^25^, coccidiosis^26^, toxoplasmosis^27–29^, sarcocystosis^30–32^, leucocytozoonosis^33^, avian malaria^34–36^, avian cholera^37^, mycobacteriosis^38,39^, trichomoniasis^40^, other bacterioses^41–43^, avian pox^44^, herpes^45^, avian influenza^46,47^, Newcastle disease^48^, eastern equine encephalitis^49^, and West Nile encephalitis^15^. However, many of these infections are known from only single cases or case clusters and affected birds often present with comorbidities, such that the importance of infection for bald eagle population health remains unclear.

Since approximately 1994, bald eagles near the lower Wisconsin River in Wisconsin, USA, have experienced a lethal and enigmatic clinical syndrome, Wisconsin River eagle syndrome (WRES)^50^. WRES, which has not previously been described in the peer-reviewed literature, has been diagnosed in most years since its initial documentation, with mortality occurring between November and early April and peaking in January and February. Affected birds generally show good body condition, suggesting acute onset, and exhibit severe neurologic deficits (weakness, incoordination, tremors, vomiting and seizures). The condition is refractory to treatment, and death or euthanasia follow shortly. WRES is characterized by hepatocellular cytoplasmic vacuolation, with vasculitis and cerebral microhemorrhages sometimes observed. The neurologic signs and hepatic pathology characteristic of WRES initially led to investigations of potential toxic causes, but no suspect chemical compounds have been identified. Similarly, testing for known pathogens, including neurotropic viruses such as West Nile virus, has failed to identify an infectious etiology.

In an effort to identify the cause of WRES, we examined bald eagle tissues archived at the Wisconsin Department of Natural Resources and the NWHC. Submissions of carcasses to these agencies included cases from eagles in Wisconsin that had died of WRES as well as eagles from Wisconsin and across the coterminous USA that had putatively died from other causes. Given the known susceptibility of raptors to viral encephalitides (e.g. West Nile encephalitis^15^) and the observation of neurologic signs and hepatic pathology in affected eagles, we investigated potential viral causes. Our analyses led to the identification of a novel hepacivirus-like virus (*Flaviviridae*: *Hepacivirus*), the type-virus of which is the globally important human pathogen hepatitis C virus (HCV)^51^. Herein we characterize the virus and describe our subsequent investigations into its distribution across the coterminous United States, its potential association with clinical disease, the population genetic structure of host and virus, and patterns of host-virus coevolution.

## Results

### Virus identification and characterization

**Figure 1:**
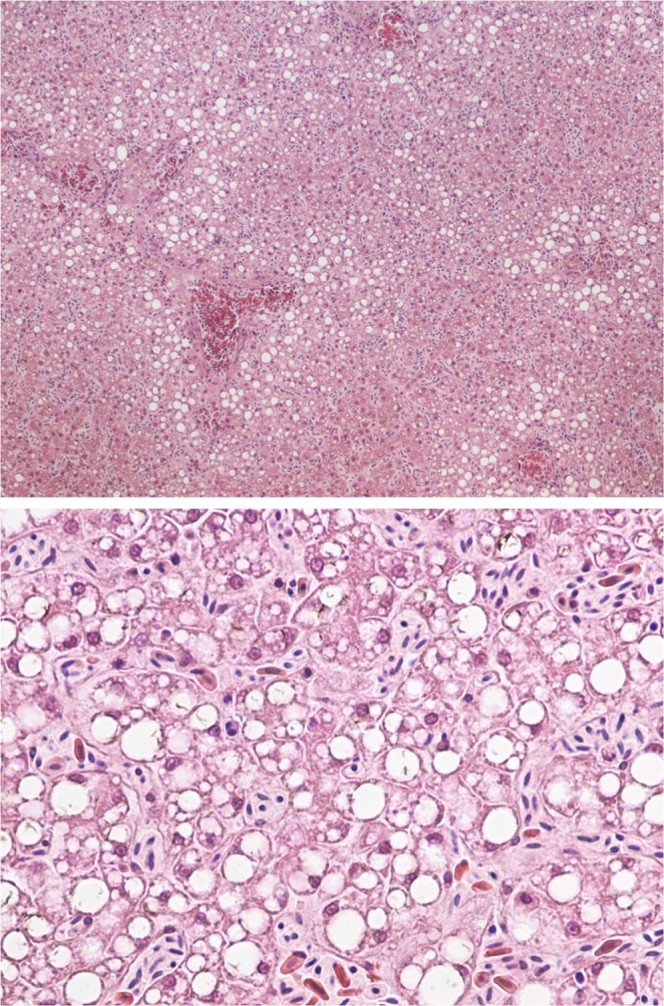
Liver from a bald eagle that died from Wisconsin River eagle syndrome stained with hematoxylin and eosin at 100x magnification (top) and 400x magnification (bottom), showing diffuse hepatocellular cytoplasmic vacuolation characteristic of this condition.

**Figure 2:**
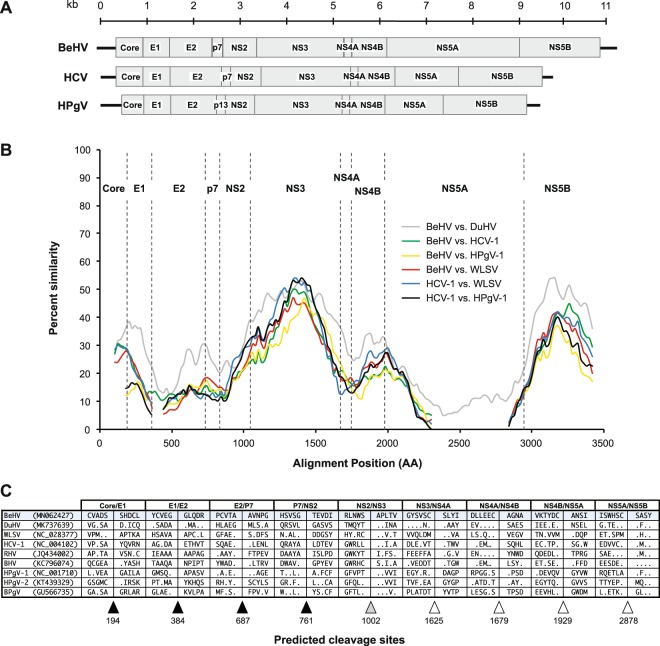
Genome organization, amino acid similarity, and polyprotein cleavage sites of bald eagle hepacivirus (BeHV) and related viruses. A) Genomic organization of BeHV, hepatitis C virus (HCV, the type virus of the genus *Hepacivirus*), and human pegivirus (HpGV; the type virus of the genus *Pegivirus*). Boxes represent mature proteins and are drawn to scale, and lines adjacent to core and NS5B proteins represent untranslated regions (UTRs). B) Sliding-window similarity plots across aligned amino acid sequences showing comparisons among BeHV, duck hepacivirus (DuHV), hepatitis C virus 1 (HCV-1), Wenling shark virus (WLSV), and human pegivirus 1 (HPgV-1). Dashed vertical lines indicate start positions of inferred viral proteins. C) Amino acid sequences of BeHV and related viruses adjacent to protease cleavage sites inferred using bioinformatic prediction. Predicted cleavage sites for signalase (black triangles), NS2-NS3 protease (gray triangle), and NS3-NS4A protease (white triangles) are indicated. Amino acid positions of cleavage sites in relation to BeHV are shown below the triangles.

**Figure 3:**
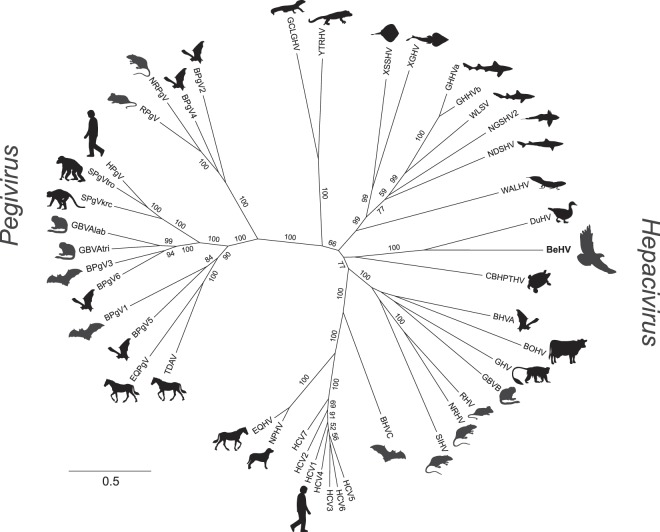
Maximum likelihood phylogenetic tree of hepaciviruses and pegiviruses, Silhouettes indicate the host in which each virus was originally described (right-facing for hepaciviruses and left-facing for pegiviruses); full virus names and GenBank accession numbers are given in Table S3. Numbers beside nodes indicate statistical confidence (percent) based on 1,000 bootstrap replicates of the data (only values ≥ 50% are shown); scale bar indicates nucleotide substitutions per site.

Next-generation sequencing of reverse-transcribed RNA from tissues of nine bald eagles that had died of WRES revealed the presence of a novel virus, which we designated bald eagle hepacivirus (BeHV; GenBank accession number MN062427). The virus was identified in serum from a bird collected on December 29, 2002, from Sauk County, Wisconsin, and the bird displayed hepatocellular cytoplasmic vacuolation characteristic of WRES (Fig. 1). The 11,019 nucleotide coding-complete viral genome sequence contained a single, 3430 amino acid open reading frame and genomic features characteristic of members of the genus *Hepacivirus* within the family *Flaviviridae* (Fig. 2A), including a model Kozak sequence (AAGAUGG) at the proposed translation initiation site. Across the genome, BeHV is most similar to duck hepacivirus (DuHV^52^) and generally more similar to HCV than to human pegivirus (HPgV), including immediately downstream of the E1/E2 junction, where homology between BeHV and other viruses is undiscernible (Fig. 2B). Similar to other members of the genus *Hepacivirus*, BeHV shows clear evidence of intrinsically disordered regions spanning the capsid protein and the 5′ half of NS5A^53^. Predicted cleavage sites are most conserved between BeHV and DuHV and show varying levels of conservation between BeHV and other hepaciviruses and pegiviruses (Fig. 2C). Phylogenetic analysis indicates that BeHV and DuHV form an avian lineage that is divergent with respect to the mammalian hepaciviruses and other hepacivirus-like viruses of non-mammalian hosts (Fig. 3). The mammalian hepaciviruses and the non-mammalian hepacivirus-like viruses together form a clade distinct from the pegiviruses (Fig. 3).

Nucleotide-level similarity within the 5′UTR, which has proven useful for inferring hepacivirus taxonomy and function^54–56^, is low between BeHV and homologous regions of HCV (50.5%) and the human pegivirus HPgV (41.8%). Minimum free energy prediction of RNA secondary structure shows this portion of the BeHV 5′UTR to exhibit three large and well-defined stem-loop (SL) structures (Fig. S1). This predicted secondary structure more closely resembles that of the 5′UTR of HCV, a member of the genus *Hepacivirus*, than the 5′UTR of HPgV, a member of the genus *Pegivirus* (Fig. S1), and it also resembles that of rodent hepacivirus^56^. However, the SL proximal to the polyprotein start codon lacks the obvious pseudoknot structure proposed for several hepaciviruses^57^, and the BeHV internal ribosome entry site (IRES) more closely resembles those of certain pegiviruses, as has been noted for DuHV^52^.

### Distribution of BeHV across the coterminous USA

Testing of 47 eagle liver tissues (which we chose to perform based on the known hepatic tropism of the hepaciviruses) from Wisconsin and elsewhere in the United States (Table S1) using metagenomics and nested rt-PCR revealed 14 additional samples to be positive for BeHV. Overall, 15/47 (31.9%) eagle liver samples were positive for BeHV, and these were collected from seven states (KS, FL, MN, ND, NE, WA and WI) out of 19 states where samples were available, with infections spanning 4,254 km of the coterminous United States (Table S1). Nested rt-PCR targeting negative-strand BeHV RNA produced amplicons of the expected size, which we confirmed by Sanger sequencing, indicating active viral replication in liver tissues.

Real time quantitative reverse transcription PCR (RT-qPCR) of RNA from liver tissues of the 15 BeHV-positive eagles yielded Ct values ranging from 26.1 to 36.4, with an overall mean of 30.26 (Fig. S2). Ct values from liver tissues of eagles from Wisconsin (Table S1) were not statistically different from Ct values of liver tissues from eagles from elsewhere (Fig. S2). The eagle in which we originally detected BeHV had a Ct value of 31.9, which was within the range of Ct values of other positive eagles (Fig. S2).

Between 1995 and 2018, the NWHC received 1188 bald eagle submissions, of which 260 (21.9%) were from Wisconsin. Of the 260 eagle submissions from Wisconsin, 28 (10.8%) were diagnosed clinically and histopathologically with WRES. No cases of WRES were diagnosed outside of Wisconsin. Of the 28 eagles diagnosed with WRES in Wisconsin, 14 (50.0%) came from counties bordering the lower Wisconsin River. Based on molecular testing of tissues from 47 eagles (described above), the prevalence of BeHV in Wisconsin was 75.0% (95% confidence interval 40.1–93.7%), whereas the prevalence of BeHV elsewhere was 23.1% (95% confidence interval 12.4–38.5%), making eagles from Wisconsin 9.4 times more likely to test positive for BeHV than eagles from elsewhere (odds ratio = 9.406, 95% confidence interval 1.684–77.79; Fisher’s exact *P* = 0.0086). Similarly, the prevalence of BeHV in eagles from counties in Wisconsin where WRES had been diagnosed was 83.3% (95% confidence interval 41.8–98.9%), whereas the prevalence of BeHV in eagles from elsewhere was 24.4% (95% confidence interval 13.7–39.5%), making eagles from counties in Wisconsin where WRES had been diagnosed 14.5 times more likely to test positive for BeHV than eagles from elsewhere (odds ratio = 14.470, 95% confidence interval 1.782–378.80; Fisher’s exact *P* = 0.0188).

### Host and virus population genetics and coevolution

**Figure 4:**
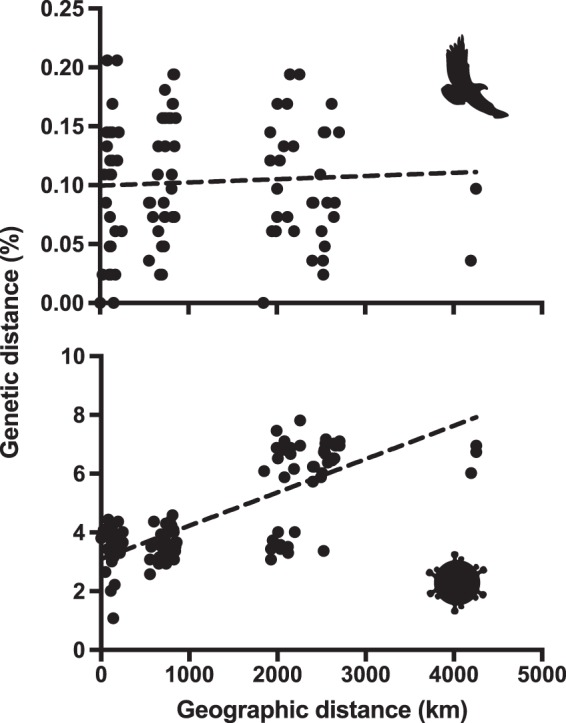
Isolation by distance for bald eagles and their viruses across the coterminous United States. Points indicate geographic-genetic distances between pairs of bald eagles (top) and bald eagle hepaciviruses (bottom); see Fig. 1 and Table S1 for details. Dashed lines are least squares regression lines.

Nucleotide-level genetic diversity (±standard error) among eagle mitochondrial DNA sequences (GenBank accession numbers MN062428-MN062562) was low (*π* = 1.03 × 10^−3^ ± 2.35 × 10^−4^). Nucleotide level genetic diversity among virus envelope gene sequences from those same eagles (GenBank accession numbers MN062563-MN062576) was higher (*π* = 4.61 × 10^−2^ ± 3.43 × 10^−3^). Analysis of isolation-by-distance revealed different patterns for hosts and viruses (Fig. 4). Mantel tests of matrix correlation indicated no statistically significant correlation between geographic and genetic distances for eagle mitochondrial DNA sequences (*r* = 0.120; 2-tailed *P* value = 0.254) but a strong and statistically significant positive correlation between geographic and genetic distances for envelope gene sequences of viruses infecting those same eagles (*r* = 0.771; 2-tailed *P* value < 0.0001). Virus pairs tended to sort into clusters of genetic similarity at discrete classes of geographic separation, revealing a spatial signal of viral population genetic substructuring across the coterminous USA that was not evident in eagle hosts (Fig. 4).

**Figure 5:**
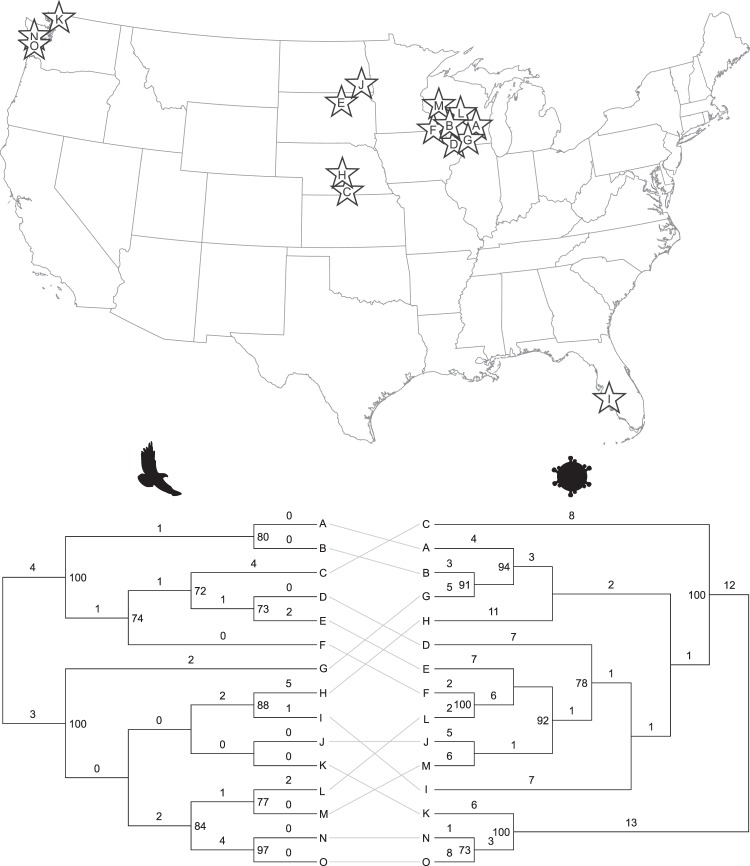
Geographic origins and cophylogenetic association of bald eagles and their viruses. Letters correspond to the locations from which eagles and their viruses were sampled, represented by stars on the map of the coterminous United States (top). For the tanglegram (bottom), numbers above branches indicate branch lengths (nucleotide substitutions per site × 10^−4^ for hosts and × 10^−2^ for viruses), and numbers beside nodes indicate bootstrap values based on 1000 bootstrap replicates of the data. Full information on eagles and viruses is given in Table S1.

Phylogenetic analyses of eagle mitochondrial DNA sequences and virus envelope gene sequences yielded different topologies (Fig. 5). Maximum likelihood cladograms (Fig. 5, bottom) were inferred from an 8,266-position alignment of concatenated mitochondrial gene sequences of eagles and a 1,395-position alignment of envelope gene sequences of viruses using the same methods described for Fig. 3. Eagle sequences showed no apparent phylogeographic pattern and branch lengths were short, whereas viral sequences showed evidence of phylogeographic clustering (e.g. viral sister taxa B and G and the clade consisting of viruses K, N and O) and branch lengths were longer (Fig. 5). Cophylogenetic analyses (Fig. 5) showed virus/host pair N and O to be sister taxa and virus/host pair A and B to be closely related in both phylogenies, but analysis of cophylogenetic association using the AxParafit algorithm^58^ with 99999 permutations revealed no statistically significant overall cophylogenetic structure between eagle and virus phylogenies (*P* = 0.139) and only one statistically significant individual association (between host/virus pairs N and O; *P* = 0.013).

## Discussion

Bald eagles are an iconic North American wildlife species that has faced myriad challenges. In the 1960s, bald eagles were nearly extirpated from the coterminous USA due to the toxic effects of DDT^3,4^. Banning of DDT in the 1970s^6^ and improved Federal protections^7,8^ allowed the species to rebound, but eagles still experience significant mortality from anthropogenic factors such as poisoning, illegal hunting, trauma, habitat loss, and antagonistic interactions with people^12^. Our results highlight two additional factors that have received comparatively little attention with respect to bald eagle conservation: infectious disease and lack of genetic diversity. We describe a novel hepacivirus-like virus, bald eagle hepacivirus (BeHV), discovered during an investigation into Wisconsin River eagle syndrome, a lethal affliction of bald eagles. We also document remarkably low genetic diversity in bald eagles across the coterminous United States, consistent with the species’ recent recovery from a population bottleneck^11,59^.

We discovered BeHV during an attempt to determine the cause of WRES, which has defied etiologic diagnosis for decades^50^, but the role of the virus in clinical disease remains uncertain. We identified the virus in the serum of a single affected eagle, but we did not find BeHV in this bird’s brain tissue or in serum or brain tissues from eight other eagles that had died of WRES. We also found BeHV in 31.9% of eagle liver samples from across the USA. BeHV viremia is therefore likely transient or intermittent, and the site of persistence is probably the liver, as is characteristic of hepaciviruses^51^. Our initial analyses focused on brain and serum were therefore based on erroneous assumptions that a neurtropic virus was involved in WRES, because of the neurologic signs observed in many affected birds^50^.

Loss of liver function can lead to hepatic encephalopathy, or accumulation of neurotoxic substances and their metabolites, which can cause severe neurologic deficits^60–62^. Outwardly, the liver pathology characteristic of many WRES-affected eagles (e.g. Fig. 1) resembles that in human patients with chronic HCV infection^63^. Cytoplasmic vacuolation in general often follows exposure to viral pathogens^64^, although this lesion is inherently non-specific and can result from non-viral causes as well. Negative-strand RT-PCR demonstrated that BeHV actively replicates in liver tissues of infected birds, but we were unable to determine whether the virus was localized at the site of hepatocellular cytoplasmic vacuolation because our attempts to use RNAscope technology^65^ to visualize BeHV in eagle livers failed due to the sub-optimal preservation conditions of field-collected specimens. Experimental infections of eagles or a suitable surrogate avian species will likely be necessary to determine the tropism of BeHV and to clarify its role in pathology and clinical disease.

We documented BeHV infection in eagles across 4,254 km of the coterminous USA, as far apart as the states of Florida and Washington. We also documented that eagles sampled from Wisconsin were 9.4 times more likely to be infected with BeHV than eagles from elsewhere, and that eagles from counties in Wisconsin where WRES had been diagnosed were 14.5 times more likely to be infected with BeHV than eagles from elsewhere. In a sample of 1188 eagles submitted to the NWHC over 23 years, WRES was diagnosed only in eagles from Wisconsin. This apparent concentration of WRES in Wisconsin (especially in counties bordering the lower Wisconsin River) may indicate locally favorable conditions for transmission or, alternatively, reporting or diagnostic bias.

Infections can weaken wildlife, making animals more vulnerable to other causes of mortality, such as predation, trauma, and starvation^66,67^. BeHV could predispose eagles to such factors. If so, region-specific co-factors could interact with BeHV to precipitate particular disease manifestations. For example, parts of the lower Wisconsin River remain ice-free in Winter, providing eagles with rare fishing opportunities and creating dense aggregations of birds that attract tourists. Eagles could transmit infections under such circumstances^14^, and indeed WRES tends to strike in the Winter months^50^. The same favorable conditions could also allow eagles to survive more readily to the end stages of infection, perhaps explaining the good body condition characteristic of WRES-affected eagles^50^. Nevertheless, positive eagles from Wisconsin had viral loads no higher than those of birds from elsewhere. Unfortunately, our sample included only moribund or dead eagles submitted to state and federal agencies, which is not a representative sample of the population of eagles at large, such that additional effort will be required to ascertain whether BeHV is causally related to WRES in the wild.

Eagles showed no evidence of isolation by distance, as might be expected from their low genetic diversity likely resulting from bottleneck effects during their near-extinction in the 1960s^59^ and their ability to fly long distances. By contrast, BeHV showed higher genetic diversity, strong evidence of isolation by distance, and distinct sorting of pairwise genetic distances into discrete geographic clusters (Fig. 4). Viral genomes (especially those of RNA viruses such as BeHV) evolve orders of magnitude more rapidly at the nucleotide sequence level than do vertebrate genes^68^. Geographic substructuring of BeHV across the coterminous United States may therefore reflect population structure in bald eagles that is not yet evident in bald eagle genomes, such as might result from subspecific or subpopulation differentiation related to migration and reproduction^8^. For example, viruses N, O and K in Fig. 4 are divergent from other viruses and also represent the only eagles analyzed from the Pacific flyway.

Eagle mitochondrial DNA and virus envelope gene sequence phylogenies were uncorrelated (Fig. 5). Because mitochondrial DNA is maternally inherited, this observation suggests that BeHV is not typically vertically transmitted. West Nile virus, another member of the family *Flaviviridae*, can be transmitted to bald eagles through feeding on infected prey and carrion, and perhaps directly from eagle to eagle, circumventing the normal vector-borne mode of transmission for this virus^14^. Bald eagles often congregate around food sources, especially in winter (when WRES most commonly occurs), where they interact aggressively with conspecifics and with other predatory birds during competition over carrion or prey items (“piracy”)^69–71^, an aspect of their natural history that famously inspired Benjamin Franklin to impugn the species as “a bird of bad moral character”^1^. Such features of eagle social behavior could predispose them to cross-species and horizontal viral transmission. The reservoir of BeHV could be bald eagles, their prey/carrion, or other species with which they interact. Although no hepaciviruses are known to be vector-borne, this possibility should also not be discounted.

The family *Flaviviridae* currently contains four genera: *Flavivirus*, *Pestivirus*, *Pegivirus*, and *Hepacivirus*^72^. Members of the genus *Flavivirus* commonly infect birds (e.g. West Nile virus) and can cause lethal disease^73^. The genus *Pestivirus* contains viruses that have so far only been found in mammals^54,74,75^. The genus *Pegivirus*, also so far found only in mammals, contains an equine virus associated with Theiler’s disease of horses^75–77^ but no other known pathogens. HPgV is nevertheless of clinical interest because it may slow disease progression in patients with AIDS and Ebola virus disease through mechanisms that remain incompletely understood^78,79^. Members of the genus *Hepacivirus* infect diverse mammals^54,75,76^, and hepacivirus-like viruses have been found in reptiles, amphibians, fishes and domestic ducks^52,54,80–82^. The hepaciviruses and pegiviruses are gaining recognition as examples of rapidly expanding viral genera that may hold important clues about the origins and pathogenesis of human and animal diseases alike^54,75,80,81^.

Our results support the notion that the diversity and host range of the hepaciviruses will likely continue to expand. BeHV and DuHV form an avian lineage of hepacivirus-like viruses that also share genomic features with the pegiviruses, suggesting an undiscovered diversity of related avian viruses that may possess similar intriguing attributes. Across the genome, BeHV is more similar to the hepacivirus type virus (HCV) than to the pegivirus type virus (HPgV), suggesting a greater affinity of BeHV for the hepaciviruses than the pegiviruses. More broadly, our results show that the mammalian hepaciviruses form a sub-clade within a diverse clade of hepacivirus-like viruses from non-mammalian hosts, and the mammalian hepaciviruses and non-mammalian hepacivirus-like viruses form a clade distinct from the pegiviruses (Fig. 3). Taxonomic subdivision of the genus *Hepacivirus* may be necessary, especially if additional discoveries support the association of clades of hepacivirus-like viruses with particular host taxonomic groups (e.g. avian, amphibian, elasmobranch)^75^.

Overall, our results show that bald eagles across the coterminous United States are infected with a novel hepacivirus-like virus that displays intriguing genomic features, epidemiologic patterns, and evolutionary history. BeHV evolution and population genetics are strikingly decoupled from those of its host, perhaps reflecting a “lag effect” due to the recent recovery of eagles from a severe population bottleneck. The extent to which BeHV represents a threat to bald eagle health and conservation will require further investigation, as will determining its origin, reservoir(s) and mode(s) of transmission. Additional studies using expanded sample sets and associated clinical data from bald eagles and other birds should add to our understanding of BeHV and its relatives. Our findings already reveal how much remains to be discovered about health and conservation in North American wildlife, even in species central to the history and cultures of the continent.

## Methods

### Ethics statement and eagle tissues

We obtained bald eagle samples from tissue archives maintained by the NWHC and the WDNR from carcasses submitted post-mortem. We used the records system of the NWHC to obtain data on numbers of eagles submitted per year, their geographic origins, causes of death, and pathologic findings. Tissues were submitted, archived, and analyzed in accordance with all local, state, tribal, and federal laws and policies, and with appropriate permits. No live animals were used in this research.

### Molecular methods

We processed serum and brain tissues from nine eagles that had died of WRES for deep sequencing and virus discovery as described previously^83,84^. Briefly, we isolated viral nucleic acids using the QIAamp MinElute virus kit (Qiagen, Hilden, Germany), converted RNA to double-stranded cDNA using random hexamers, and prepared libraries for sequencing on an Illumina MiSeq intrument (V3 chemistry, 600 cycle kit; Illumina, San Diego, CA, USA) using the Nextera XT DNA sample preparation kit (Illumina, San Diego, CA, USA). We analyzed sequence data using CLC Genomics Workbench version 11.0 (CLC bio, Aarhus, Denmark), first trimming low-quality bases (phred quality score < 30) and discarding short reads (<75 bp). We then analyzed unassembled reads and contiguous sequences derived from *de novo* assembly of raw reads for nucleotide-level (blastn) and protein-level (blastx) similarity to viruses in GenBank as previously described^83,84^.

Based on the known hepatic tropism of the hepaciviruses, we next examined archived liver tissues of 47 birds (fresh frozen at −80 °C), including the aforementioned juvenile eagle, of which eight were from Wisconsin (three of these from counties bordering the lower Wisconsin River) and 39 were from elsewhere (Table S1). From each sample, we collected 6 mm sterile punches (60 mg), suspended them in 1 mL of 1x Trizol in tubes with 2.38 mm metal beads (Qiagen, Hilden, Germany), and homogenized them in three 20-second cycles with a Mini-Beadbeater-96 instrument (Biospec Products, Bartlesville, OK). We then centrifuged the tubes at 12,000 × *g* for 5 minutes at 4 °C and supplemented 100 µL (~6 mg tissue equivalent) of supernatant with 900 µL of Trizol. We performed phase separation in 2 mL Phase Lock Gel Heavy tubes (5PRIME, Gaithersburg, MD) and purified nucleic acids from the aqueous phase using the RNA Clean & Concentrator-5 kit (Zymo Research, Irvine, CA). We then conducted deep sequencing as described above.

To test eagle liver samples for BeHV, we designed a nested RT-PCR using primers annealing to the highly conserved NTPase region of the viral NS3 gene (Table S2). We performed RT-PCR using the OneTaq RT-PCR kit (New England Biolabs, Ipswich, MA) followed by the KAPA HiFi HotStart PCR kit (KAPA Biosystems, Wilmington, MA). We included primers at 400 nM each and conducted RT-PCR as follows: 53 °C for 30 minutes, 94 °C for 2 minutes; 94 °C for 15 s, 56 °C for 30 s, and 68 °C for 2.5 min for 40 cycles; and a terminal extension step at 68 °C for 5 min. We then conducted 35 cycles of nested PCR using 1 µl of external PCR product as template and the same conditions described above, but omitting the initial reverse transcription step and with an annealing temperature of 58 °C. We visualized amplicons on 2% agarose gels stained with ethidium bromide and purified them using the Zymoclean Gel DNA Recovery Kit (Zymo Research, Irvine, CA). We then prepared amplicons for sequencing using the Nextera XT DNA sample preparation kit (Illumina, San Diego, CA), followed by sequenced on an Illumina MiSeq (MiSeq Reagent Kit, v3, 150 cycles, Illumina, San Diego, CA).

To assess viral genetic variation, we amplified and sequenced the viral envelope gene in all positive tissues using nested RT-PCR assay (Table S1) and the same reagents described above. We included primers at 400 nM each and completed RT-PCR as follows: 50 °C for 30 minutes, 94 °C for 1 minute; 40 cycles of 94 °C for 15 s, 56 °C for 30 s, and 68 °C for 1.5 min; and a terminal extension step at 68 °C for 5 min. We conducted 35 cycles of nested PCR using 1 µl of external PCR product as template as described above, but omitting the initial reverse transcription step. We then visualized, purified, and sequenced PCR products as described above.

To assess eagle genetic variation and to provide host relatedness data for cophylogenetic analyses, we assembled sequences of 9 mitochondrial genes (control region, ND2, COX1, COX2, ATP6, COX3, ND4, ND5, and CYTB) from deep sequence data generated from liver tissues of all BeHV-positive eagles liver samples sequenced and analyzed as described above. We then concatenated the resulting sequences for population genetic and phylogenetic analyses.

To assess viral replication in liver tissues, we amplified negative-strand (replicative-intermediate) RNA following the methods of Lin *et al*.^85^. We used the same primers, reagents and cycling conditions for nested RT-PCR described above, except that we appended the primer annealing to negative-strand RNA during reverse transcription (BeHV-NS3-EX-F3869) with a 5′ blocking sequence (Table S2). During flanking RT-PCR, we added primer WRBEV-R4084 following reverse transcription and a reverse transcriptase inactivation step. We then used the 5′ tag sequence (Table S2) as the forward primer and primer BeHV-R4017 as the reverse primer to preclude amplification of viral RNA that was either self-primed or primed by endogenous oligonucleotides during reverse transcription. We then verified amplicons by Sanger sequencing.

To assess relative viral loads, we conducted RT-qPCR using primers (Table S2) designed to anneal to regions conserved among all BeHV envelope gene sequences (determined as described above) and a ZEN double quenched probe (Integrated DNA Technologies, Coralville, IA). We ran reactions in triplicate on a BioRad CFX96 real-time system mounted on a C1000 thermocycler (BioRad, Hercules CA) using the GoTaq Probe 1-Step RT-qPCR System (Promega, Madison, WI) according to the manufacturer’s recommendations, with 2 µl RNA (extracted from 60 mg tissues, as described above) as input. We calculated viral loads as the Ct value averaged across three rtq-PCR replicates.

To visualize BeHV RNA *in situ*, we attempted to apply RNAscope technology^65^ to histologic sections of eagle livers using RNAscope double “Z” oligonucleotide probes designed to hybridize to the BeHV polymerase gene; however, all attempts failed, likely because of the degraded nature of RNA in tissues from carcasses collected under field conditions.

### Viral genome analyses

We determined the putative translation initiation codon for the BeHV open reading frame using the ATGpr prediction server^86^, we used SignalP^87^ to identify signal peptide sequences, and we used the IRESPred web server^88^ to characterize the BeHV IRES. We also conducted analyses of core and NS5A protein sequences to identify intrinsically disordered regions (IDRs) and sites within IDRs with capacity to undergo disorder-to-order transitions for binding interactions using ANCHOR^89,90^. Because of previous work showing that the 5′ untranslated region (UTR) of the hepaciviruses is informative for viral taxonomy and prediction of function^55^, we analyzed homologous 3′ ends of this feature in BeHV, HCV and HPgV to predict RNA secondary structure using the mfold Web Server^91^ and the RNAfold algorithm executed on the Vienna RNA Websuite server^92,93^.

### Phylogenetic and evolutionary analyses

To infer the phylogenetic position of BeHV, we compiled nucleotide sequences of complete viral polyprotein genes available in Genbank representing known lineages within the hepaciviruses and pegiviruses. We then generated codon-based sequence alignments using the PRANK algorighm^94^ with the Gblocks algorithm^95,96^ applied to remove poorly aligned regions using TranslatorX^97^. We inferred phylogenies from the resulting 3,579-position nucleotide sequence alignment using PhyML^98^, with the model of molecular evolution (GTR + Γ + I) estimated from the data^99^ and 1,000 bootstrap replicates to assess statistical confidence in clades, and we displayed trees using FigTree^100^. To compare BeHV and select hepacivirus-like viruses, hepaciviruses, and pegiviruses, we aligned full viral genomes as described above and examined variation in amino acid-level similarity across the length of the alignment using the sliding-window method implemented in SimPlot^101^.

To assess host and viral genetic diversity, we calculated nucleotide diversity (*π*^102^) from aligned eagle and virus gene sequences using the computer program MEGA7^103^. To assess isolation by distance, we computed pairwise geographic distances between centroids of the US county of origin from which each eagle was collected using Geographic Distance Matrix Generator^104^. We then computed uncorrected pairwise genetic distances between eagles (mitochondrial gene sequences) and viruses (envelope gene sequences) and compared each to pairwise geographic distances using Mantel tests of matrix correlation^105^ with 10,000 permutations of the data to assess statistical significance, implemented in the APE package in R^106^.

To assess host-virus coevolution, we conducted cophylogenetic analyses. We generated codon-based sequence alignments of eagle concatenated mitochondrial DNA sequences and virus envelope gene sequences and inferred maximum likelihood phylogenies as described above. We then constructed tanglegrams using Dendroscope^107^ and tested for overall phylogenetic congruence and the significance of individual host/virus associations using the AxParafit algorighm^58,108^ implemented in CopyCat, version 2.04^109^.

## Supplementary information

Supplementary Figure S1

Supplementary Figure S2

Supplementary information

Supplementary information

Supplementary information

## Data Availability

All data generated during the current study are available in GenBank (accession numbers MN062427- MN062576) or are included in this published article and its Supplementary Information files.
