# Supplementary material for: Multidecade Mortality and a Homolog of Hepatitis C Virus in Bald Eagles (Haliaeetus leucocephalus), the National Bird of the USA
Source: Sci Rep. 2019 Oct 18;9:14953. doi: 10.1038/s41598-019-50580-8 (PMC6802099; doi:10.1038/s41598-019-50580-8)
Supplement: Supplementary file 1 — Supplementary Figure S1 [file 41598_2019_50580_MOESM1_ESM.pdf]

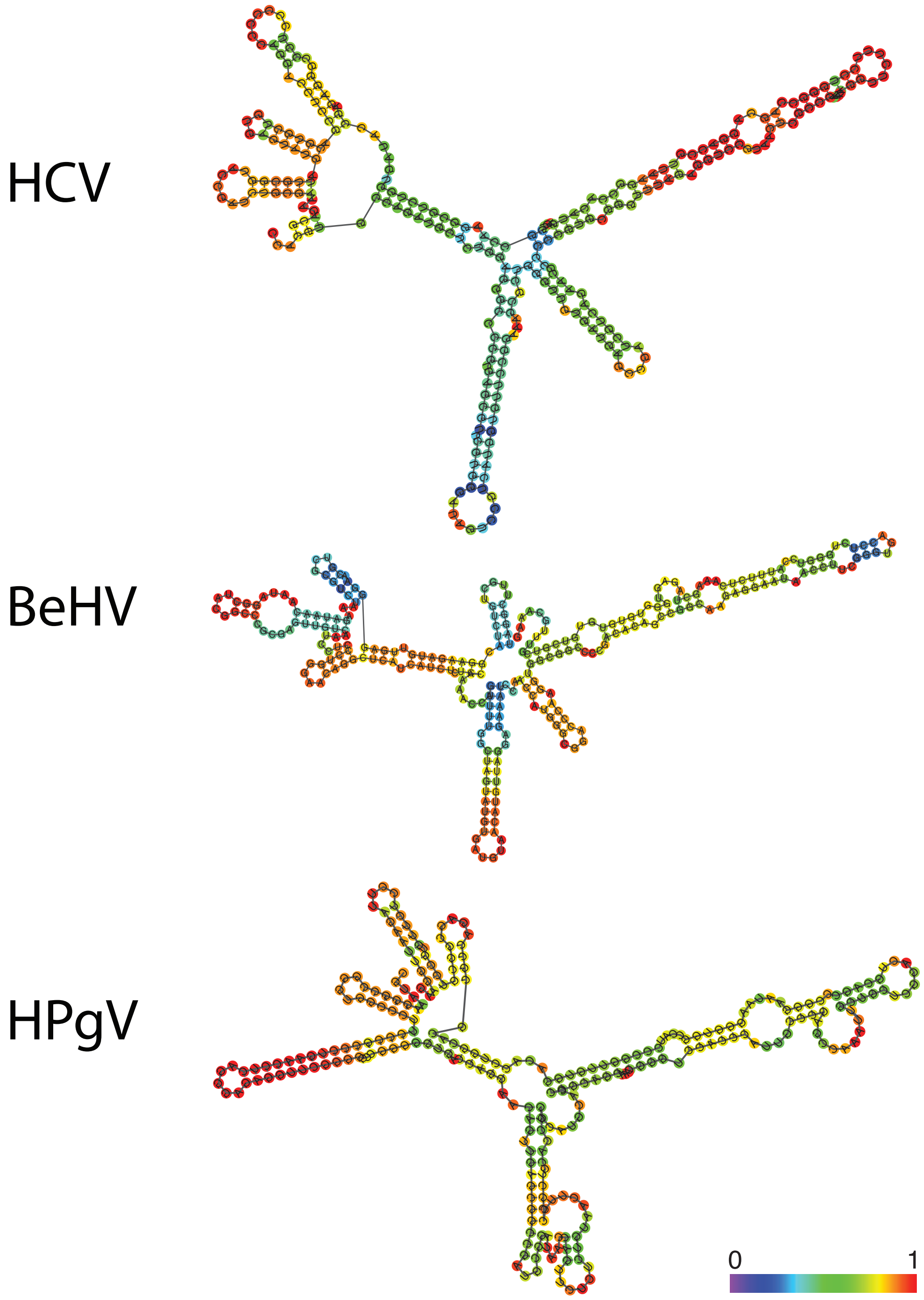

Supplementary Figure S1: Predicted RNA secondary structures of homologous regions of 5' untranslated regions of hepatitis C virus, genotype 1 (HCV; GenBank accession number NC_004102), bald eagle hepacivirus (BeHV; GenBank accession number MN062427), and the human pegivirus GB virus C (HPgV; GenBank accession number NC_001710). Structures are minimum free energy predictions (-110.12, -87.67, and -118.62 kcal/mol, respectively) generated by the RNAfold algorithm on the Vienna RNA Websuite server[92,93]; colored scales indicate base-pair probabilities.
