# Supplementary material for: Multidecade Mortality and a Homolog of Hepatitis C Virus in Bald Eagles (Haliaeetus leucocephalus), the National Bird of the USA
Source: Sci Rep. 2019 Oct 18;9:14953. doi: 10.1038/s41598-019-50580-8 (PMC6802099; doi:10.1038/s41598-019-50580-8)
Supplement: Supplementary file 2 — Supplementary Figure S2 [file 41598_2019_50580_MOESM2_ESM.pdf]

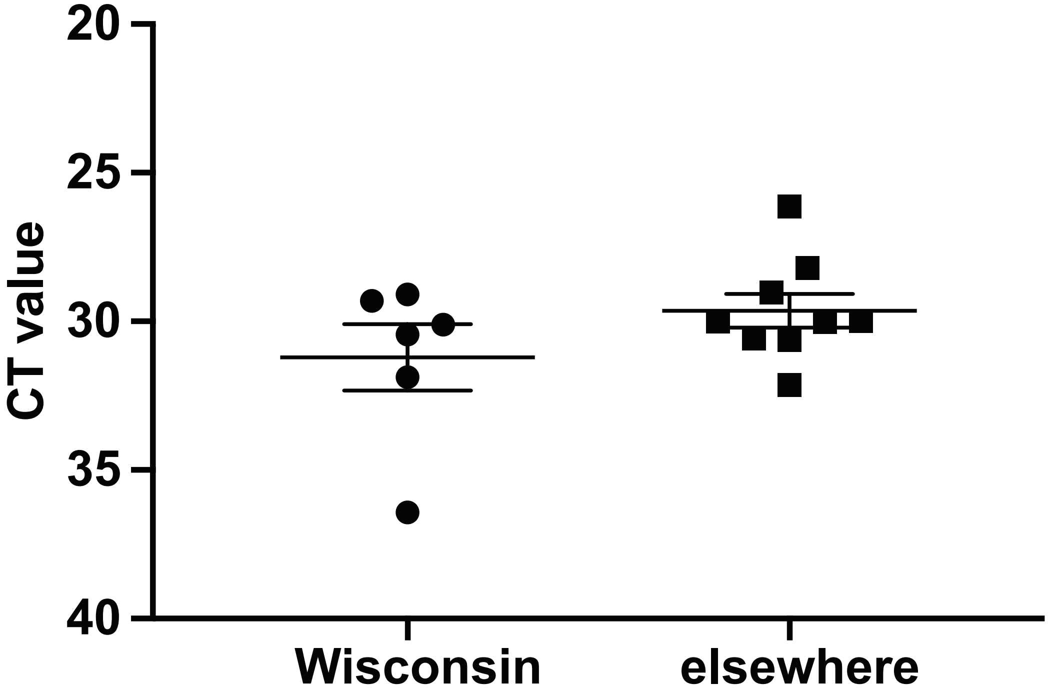

Supplementary Figure S2: Relative loads of bald eagle hepacivirus (BeHV) in bald eagle liver tissues. Ct values are averages across triplicate quantitative real-time reverse transcription PCR assays. Lower Ct values indicate higher relative viral loads, lines indicate means, and error bars indicate standard errors of the mean. Values are shown for six eagles from Wisconsin compared to nine eagles from elsewhere (see Table S1); the difference is not statistically significant (t=1.376; df=13; P=0.1920).
