# Supplementary material for: Multidecade Mortality and a Homolog of Hepatitis C Virus in Bald Eagles (Haliaeetus leucocephalus), the National Bird of the USA
Source: Sci Rep. 2019 Oct 18;9:14953. doi: 10.1038/s41598-019-50580-8 (PMC6802099; doi:10.1038/s41598-019-50580-8)
Supplement: Supplementary file 4 — Supplementary information [file 41598_2019_50580_MOESM4_ESM.docx]

**Table S2**. Oligonucleotide primers and probes used in the study

| *Name** | *Sequence (5'-3')* | *Amplicon size (bp)* |
| --- | --- | --- |
|  |  |  |
| *Primers for diagnostic nested PCR of the BeHV NS3 gene* | | |
| BEHV-NS3-EX-F3869† | GATGTAGTGCTTTGTGATGAGTGTCA | 262 |
| BEHV-NS3-EX-R4084 | CCTTTGAACCACAAAAGATGACGTG |  |
| BEHV-NS3-IN-F3918 | GCATTGGTACTGTGCTCACG | 138 |
| BEHV-NS3-IN-R4017 | CCACTTCTCCCTCGTCAGTC |  |
|  |  |  |
| *Primers for nested PCR and seqeuncing of the BeHV envelope gene* ‡ | | |
| BEHV-ENV-EX-F491 | TCCCTTCTTGTGTGGCTGAT | 1550-1676 |
| BEHV-ENV-EX-F525 | TCTCGTCAATGGAACTTGGA |  |
| BEHV-ENV-EX-R2074 | GAGCATAAACCCAAGCAAGC |  |
| BEHV-ENV-EX-R2166 | AAGGGCTAAAGCAGGGTAGC |  |
|  |  |  |
| BEHV-ENV-IN-F582 | GCTGTCACACATGGAAGTGC | 1348-1493 |
| BEHV-ENV-IN-F644 | CGGTTCCTTGGTTCATGACT |  |
| BEHV-ENV-IN-R1991 | GCTGTCACACATGGAAGTGC |  |
| BEHV-ENV-IN-R2074 | GAGCATAAACCCAAGCAAGC |  |
|  |  |  |
| *Primers and probe for real time quantitative PCR of the BeHV envelope gene* | | |
| BEHV-QRT-F299 | TTTTCCAAGCTCTCGCCGATAG | 146 |
| BEHV-QRT-R444 | CCTACCAGCAGCTAGATAGAGTATGA |  |
| BEHV-QRT-PRB | /56-FAM/CACTGTTCC/ZEN/AATAGGCTTGTTTAGGTTGATTG/3IABkFQ/ | |
|  |  |  |

*Numbers included in primer names indicate the nucleotide position to which the 5' base of each primer anneals within the BeHV polyprotein open reading frame.

†This primer was appended with tag sequence 5'-AAGCAGTGGTATCAACGCAGAGT-3' during negative strand PCR to assess of viral replication; see text for details.

‡Various combinations of external and internal primers were used to obtain envelope gene sequences from all BeHV-positive tissues.
